# Why Health and Social Care Support for People with Long-Term Conditions Should be Oriented Towards Enabling Them to Live Well

**DOI:** 10.1007/s10728-016-0335-1

**Published:** 2016-11-28

**Authors:** Vikki A. Entwistle, Alan Cribb, John Owens

**Affiliations:** 1 Health Services Research Unit, University of Aberdeen, Health Sciences Building, Foresterhill, Aberdeen, AB25 2ZD Scotland, UK; 2 School of Education, Communication and Society, King’s College London, Waterloo Bridge Wing, Franklin-Wilkins Building, Waterloo Road, London, SE1 9NH UK

**Keywords:** Capabilities approach, Chronic conditions, Professional-patient relations, Patient participation, Quality of life, Person centred care

## Abstract

There are various reasons why efforts to promote “support for self-management” have rarely delivered the kinds of sustainable improvements in healthcare experiences, health and wellbeing that policy leaders internationally have hoped for. This paper explains how the basis of failure is in some respects built into the ideas that underpin many of these efforts. When (the promotion of) support for self-management is narrowly oriented towards educating and motivating patients to adopt the behaviours recommended for disease control, it implicitly reflects and perpetuates limited and somewhat instrumental views of patients. It tends to: restrict the pursuit of respectful and enabling ‘partnership working’; run the risk of undermining patients’ self-evaluative attitudes (and then of failing to notice that as harmful); limit recognition of the supportive value of clinician-patient relationships; and obscure the practical and ethical tensions that clinicians face in the delivery of support for self-management. We suggest that a focus on enabling people to live (and die) well with their long-term conditions is a promising starting point for a more adequate conception of support for self-management. We then outline the theoretical advantages that a capabilities approach to thinking about living well can bring to the development of an account of support for self-management, explaining, for example, how it can accommodate the range of what matters to people (both generally and more specifically) for living well, help keep the importance of disease control in perspective, recognize social influences on people’s values, behaviours and wellbeing, and illuminate more of the rich potential and practical and ethical challenges of supporting self-management in practice.

## Introduction

The concept of support for self-management continues to attract significant interest from health policy leaders internationally as increasing numbers of people are living with long-term conditions. Support for self-management has been defined by the Institute of Medicine as “the systematic provision of education and supportive interventions by health care staff^1^ to increase patients’ skills and confidence in managing their health problems” [23]. Particularly in the context of financial constraints brought about by the politics of austerity and sustained action towards institutional ‘efficiency savings’, support for self-management has been seen as a means of improving health care while limiting health service costs. However, it is also viewed as a practice that involves staff relating to patients in ways that should generate positive experiences of healthcare, empowering them as partners and enhancing their wellbeing or overall quality of life.

Yet the simultaneous achievement of these multiple ambitions seems far from widespread. Although the shortfall might in part be attributed to health services and professionals failing to implement appropriate forms of support for self-management, this is hard to judge because questions about what constitutes appropriate support (or what support would have to look like to fulfil the multiple ambitions) have received insufficient critical attention.

In this paper, we explain how conceptual-theoretical problems contribute to the practical shortcomings of at least some efforts to promote and deliver support for self-management, particularly in terms of empowering patients as partners and enhancing their experiences of healthcare and their health, wellbeing and overall quality of life.^2^ We also outline one promising solution.

Four sections follow this introduction. “Support for self-management: policy ambitions and practical shortfalls” summarises the current situation. “Support for self-management: conceptual issues” then illustrates how both the purpose and the means of support for self-management can be more narrowly and more broadly conceived, explains why narrower ways of thinking can be problematic and argues that ambitious policy expectations for the concept demand broader thinking about both the purpose and means. “A capabilities approach and its potential for theorizing support for self-management” introduces the key ideas of a capabilities approach and outlines how it can be used to develop an account of support for self-management with the required breadth. It highlights the theoretical potential of the approach to support transformative and humanistic approaches to practice. Finally the “Discussion” reviews the case we have made and suggests directions for further practical and analytic work.

## Support for Self-Management: Policy Ambitions and Practical Shortfalls

In recent decades, a profound enthusiasm for “support for self-management” has been evident in health policy internationally [11, 23, 58]. Interest in the concept emerged in large part from questions about how health services could address, in sustainable ways, the needs of the growing numbers of people living with long-term conditions [6, 55, 56]. It reflects recognition that: people with long-term conditions have to get on with them somehow, and are involved on a daily basis in, for example, dealing with symptoms, attending to their diet and exercise, and taking medicines; developments in healthcare and information technology have increased the scope for people to monitor and treat their conditions outside formal health care facilities and without the direct involvement of health professionals; and support from health services and others could enhance the effectiveness of people’s efforts to help themselves [23, 28, 30].

But the current policy enthusiasm for support for self-management is based on more than a concern to improve people’s condition-management and reduce the kinds of demands on health services that have been cultivated by delivery models designed to deal with acute conditions. The support health professionals provide for self-management is also often expected to improve people’s broader wellbeing or quality of life, and to be consistent with so-called ‘patient-centred’ or ‘person-centered’^3^ approaches to healthcare and (so) a means of improving people’s experiences of healthcare [3, 25, 45]. Support for self-management is linked with ideas about service providers moving away from negative views of people with long-term conditions as deficient, needy and passive recipients of health care to more positive views of them as active partners or asset-bearing co-producers who themselves contribute to the solutions to their health problems [2–4, 12]. It is advocated with the intention that it will be responsive to individuals and somehow respectful of their autonomy, and is associated with notions of confidence-building, empowerment, choice and control [11, 29, 35].

Important questions need to be asked about whether and how all the high expectations evident in advocacy of support for self-management can possibly be achieved in practice. Currently, many health service organizations, delivery teams and professionals acknowledge the importance of support for self-management and aspire or claim to provide it. A plethora of training initiatives and resources have been developed, and various commercial and third sector providers offer supplementary or complementary self-management programs, tools and resources for patients [21, 39, 52]. But despite some strikingly positive reports of what some forms of self-management support have achieved in some outcome domains for some people, efforts to enhance support for self-management, and especially that delivered by health professionals in the context of routine service provision, have often proven difficult to implement and/or have not consistently generated the positive outcomes anticipated [8, 24, 26, 59]. Not only are ambitions for behaviour change and condition-control not widely achieved or sustained, but practices associated with support for self-management sometimes seem to undermine rather than enhance people’s experiences of healthcare, and to disempower rather than empower people as they use health services. Studies that have examined how health professionals go about supporting self-management in practice report (among other problems) examples of: authoritarian behaviours; under-estimation of people’s knowledge and aspirations; attitudes and actions that dismiss and undermine people’s reasonable efforts to help themselves; and negative, moralistic judgementalism against patients who do not comply with professionals’ advice or achieve condition-control targets [19, 33, 36, 41, 50].

A number of ideas have been advanced to develop the theoretical underpinnings of support for self-management. Most notably, insights from theories of learning and behaviour change have fostered interest in: looking beyond people’s health-related knowledge to their skills and self-efficacy for recommended health-related behaviours; assessing and responding to people’s motivation or state of ‘activation’ for condition-management; and personalized care planning (incorporating patient-led goal setting and reviews of progress towards goals) [20, 29, 40]. Despite these theoretical advances, it remains unclear how, in practice, support for self-management can simultaneously contribute positively to healthcare experiences, health, wellbeing and overall quality of life.

Here, while acknowledging that there are likely multiple reasons for shortfalls in practice and achievement relative to these policy ambitions, we focus on conceptual and theoretical contributions to the problems.

## Support for Self-Management: Conceptual Issues

‘Support’ is a rich and multi-faceted concept. Most dictionaries offer a range of definitions of support that include terms such as ‘encourage’, ‘offer assistance’, ‘accept’, ‘hold up’, and ‘stop from falling’. Reflections on the kinds of support provided by parents, friends and peers further indicate that a plurality of practices can be intended and experienced as supportive, and that for many and complex reasons the intent and the experience of support do not always coincide.

This richness and complexity is rarely evident in the policy advocacy of, or development and evaluation of interventions to promote, health service support for self-management. Although some statements are often in principle quite flexible about what might count as support (as, for example, the relatively open and somewhat circular mention of “supportive interventions” in the Institute of Medicine definition), guidance and interventions for professional practice tend to focus on activities associated with instructional education (informing, developing skills and enhancing self-efficacy for particular behaviours) and motivation (explaining the importance of biomedical risk factors, setting goals and monitoring progress). A current enthusiasm for measuring patient activation also fosters a focus on knowledge, skills and confidence for professionally recommended behaviours [20].

Without implying that health services and professionals should offer the same kind of support as parents, friends or peers, we suggest that further reflection is warranted on the scope of support that they can (and should) offer people with long-term conditions. In the following sub-sections we illustrate how both the purposes of support for self-management and the means by which such support is (intended to be) provided and achieved can be more narrowly and more broadly conceived. We then explain why narrower conceptions are problematic, and argue that health services and professionals need broader conceptions of both purpose and means if they are to be in a position to respect and enable people and to improve experiences of health care and overall quality of life.

### The Purpose of Support for Self-Management

Putting aside, as noted above, the policy purpose of reducing the demands on, and public costs of, formal health care provision, we now consider the question of what health service support for self-management is (or should be) trying to help people with long-term conditions achieve. This question is rarely critically examined, but the built-in answer of “self-management” needs some unpacking, and the easy consensus that can be gained with answers such as “better health or wellbeing” can hide problematic variants among the many interpretations that are possible within these.

In health service contexts, self-management usually refers to what people with health condition(s) do for themselves, perhaps with the help of family and friends, but in contrast to what health professionals do for them [11]. Yet policy ambitions for support for self-management go beyond shifting work from health systems and professionals to patients and their families, and efforts are usually made to reassure patients that self-management does not mean doing without professional input altogether [28]. Once it is recognized that people with long-term conditions have to manage them somehow, the purpose of support seems to be to enable them to manage better. This then begs the question of what counts as managing better. The possible answers have significant practical (and ethical) implications.

We have noted previously an important distinction between helping people to manage their health conditions well and helping people to manage well with their health conditions [36]. When the focus is on the better management of health conditions, support is usually biomedically framed and relatively narrowly oriented to symptom and disease control. It aims typically to help people to slow the progression of disease, reduce the risk of complications and maximize length of life with as little disease as possible. There is often a strong emphasis on encouraging people to monitor symptoms and biomedical markers (e.g. blood pressure and blood glucose levels) and to adopt behaviours recommended to regulate these. In contrast, when the purpose of support is understood in terms of helping people to manage better with their long-term conditions, disease control can still be of interest, but services work with more expansive and more flexible aspirations for health, wellbeing and quality—not just length of life.

### The Means of Support for Self-Management

When considering the means of support (how support is enacted), we suggest it is important to examine the aspects of a person’s (potential) state, agency or action that support is intended to bolster. These will usually be connected, both conceptually and in practice, to more ultimate (even if implicit) ideas about the intended purpose of support (as outlined above): a particular view of purpose will suggest that particular aspects of a person’s state, agency or action are more salient.

Many contemporary approaches enact support for self-management with an instructional-educational emphasis, and are targeted relatively narrowly to perceived deficits in people’s knowledge and skills. These approaches often view the means of support in terms of advice pertaining to diet, exercise, biomedical monitoring and/or medication regimes. The repertoire of means for supporting self-management has been broadened to some extent with attention to perceived shortfalls in people’s self-efficacy and motivation, and the associated introduction of various forms of confidence-building practices, motivational interviewing and personalized care planning. But the effective scope of these can be circumscribed by a continued focus on disease control and length of life goals [8] and limited by the neglect of more social constraints on people’s behaviours [16, 48].

Attention to the socio-economic and socio-cultural underpinnings of patients’ states, agency and action, and recognition that people’s abilities are sometimes significantly constrained by their practical and relational circumstances, bring a broader set of potential means of support into view. This includes, for example, the reduction or removal of financial charges for recommended medications and biomedical self-monitoring equipment, and initiatives to address the root causes of long-term food insecurity.^4^

Attention to people’s emotional wellbeing and autonomy, and to the factors that influence these, can also increase the range of recognized means of support for self-management. In particular, it can highlight the potential significance of health professionals’ communicative behaviours and relational (interpersonal) attitudes, which people can experience as more or less supportive—or, indeed, as undermining of how they feel in and about themselves. It suggests that health professionals can sometimes support people by, for example: acknowledging their suffering and the difficulties of accepting and dealing with the various implications of long-term conditions [18, 22]; providing the kind of “moral support” that reassures someone that they are not alone, are being taken seriously, and are not being unfairly and negatively judged; letting someone know that they are “there for you” (which does not necessarily imply being personally available 24/7); and communicating in the kinds of respectful, caring and affirmative ways that help people to retain/develop a valued self-identity, personal narrative and sense of meaning in their life in the face of a new diagnosis or the ongoing challenges and/or deterioration of lifelong conditions [15, 27].

These ways of behaving and relating can support people in ways that go beyond (or do not involve) working directly to enable them to take a particular action or achieve a particular goal, but they have potential to leave people feeling better in or about themselves and to strengthen important aspects of their autonomy [16, 31, 35].

### Practical and Ethical Significance of Narrower and Broader Conceptions

The above characterizations inevitably raise some evaluative questions about the implications of adopting narrower or broader ideas about the purpose and means of support. We now consider which ideas are better, and argue that only broader conceptions can underpin the promotion of support that respects and enables patients as human moral agents.^5^ We start by noting that while narrower views of purpose reflect some important considerations, they can also generate significant problems.

Supporting people to contribute to condition-management in the disease control sense can be extremely valuable in situations where people’s own actions can help avoid preventable outcomes that significantly undermine length and/or quality of life. Diabetes is perhaps the paradigm example of this: people strongly value, for example, not having hypo- or hyper-glycemic emergencies in the short term and not going blind, having feet/legs amputated, or being disabled by a stroke in the longer term, and most would rather (than not) be offered support to take action to avoid these complications. However, matters become much more complicated once we look beyond paradigm conditions or consider particular cases in greater depth.

There are several key considerations here. First, the behavioural regimes that are recommended for disease control, especially in the combinations that occur for people with multiple conditions, are not consistently effective (not everyone who acts as advised will achieve or sustain target biomedical goals) and they are not always particularly (or proportionately) feasible, acceptable or achievable in the first place [32].

Second, disease control is not the only thing that matters for health and wellbeing. Studies show that people with long-term conditions are (sometimes more) concerned about having a normal life, maintaining social roles and respect, and maintaining or regaining identity and emotional balance [1, 5, 7, 53]. A narrow focus on disease control can tend to squeeze these concerns out from consultation agendas.

Third, the narrow means of support that are used to encourage patients to act as recommended for disease control can become harmful in several respects. A focus on what are perceived to be patients’ psychological-behavioural deficits (as judged against biomedical ideals), combined with an emphasis on the evidence provided by biomedical and clinical epidemiological research, tends to reinforce (as well as reflect) a positioning of health professionals as experts. Some professionals use this in ways that introduce or perpetuate rather authoritarian hierarchical relationships that tend to preclude patients from functioning effectively as partners in their care [33]. Further, when health professionals hold and express negatively judgmental attitudes towards patients who do not conform to disease control ideals (including for example, assumptions that they do not care about their own health or that deteriorating biomarkers give lie to their claims about their behaviours) they can leave people feeling anxious, hopeless, unnecessarily guilty, hard done to and disrespected [14, 50, 51]. Any undermining of patients’ self-evaluative attitudes and sense of identity (especially their sense of themselves as self-authorised), as well as of their scope for self-governance and self-determination, can be understood as undermining of their autonomy [31, 34].

Fourth, when healthcare systems or professionals view disease control as the main purpose of support for self-management, they at least implicitly position patients as means to that end. In effect, they are harnessing patients to work towards biomedical goals. Of course many people are keen to do what they can to work to limit disease progression and lengthen their lives, and most healthcare professionals aspire to work for the good of their patients and resist relating to them as mere means. Nonetheless, narrow views of the purpose of support for self-management can tend to foster instrumental views of patients, which in turn tend to foster disrespectful practices.

Fifth, narrow views of purpose have no built-in protection against the possibility that actions carried out in the name of support for self-management will, in practice, impair people’s autonomy, identity or other things that matter for their experiences of healthcare and for their overall wellbeing or quality of life. These kinds of harms tend to be neglected within narrow conceptions because they are not recognized as part of the purpose of support.

Sixth, some of the negative implications of narrow views of the purpose of support for self-management, (including potential harms to emotional wellbeing and autonomy, are perhaps less likely to be manifest among people whose high levels of education, material affluence and/or knowledgeable and influential social networks render the behaviours recommended for management of their conditions relatively straightforward and perhaps increase their resilience to off-putting comments. Narrow views are thus potentially likely to exacerbate social inequalities in experiences of healthcare and wellbeing.

We note that the promotion of potentially broader means of support is unlikely to suffice to avoid the problems that can arise with narrow views about the purpose of support. Unless a broad overall purpose is articulated as well, communication strategies which could be enacted in ways that reflect and encourage respect for and enablement of patients as human moral agents can too-readily be enacted in ways that co-opt them back to relatively restricted biomedical ambitions. This is illustrated by the example of health professionals who take up the idea of personalized care planning with patient-led goal setting, but do this within a biomedical frame. As mentioned above, they might offer patients a menu of potential areas of behaviour change and encourage them to develop their own goals, but their biomedical framing constrains the scope for patients to suggest goals in more personally relevant domains [8].

Broader ideas about the purpose of support for self-management can signal more directly that support should contribute towards patients’: experiences of being respected and enabled as moral agents; scope to function as value-determining partners in their care, and; overall health, wellbeing and quality of life.

The idea of couching the purpose of support for self-management in terms of enabling people to live (and die)^6^ well with their long-term conditions [16, 36] seems particularly promising as a broad statement of purpose. It brings the person with long-term conditions more fully into view as an active moral agent, and one whose view of what counts as living well matters. It can incorporate aspects of healthcare experience, health, wellbeing and quality of life that go beyond those impacted in a knock-on kind of way by disease control.

Broader conceptions of the means of support can also reflect a more theoretically robust view of a person, give more recognition to the importance of relationships between health services or professionals and patients, and do more to illuminate the potential of these relationships to bolster (or undermine) people’s capacity and scope for autonomous agency.

We note as well, however, that taking a broad view of purpose can raise some practical and ethical challenges in support for self-management. It prompts important questions about the scope of health services’ and health professionals’ remits and also about the relative priority of the various aspects of healthcare experience, health, and wellbeing or quality of life that it encompasses. We consider these further in the Discussion.

In the following section, we introduce a capabilities approach and outline its potential for the development of a broad account of support for self-management.

## A Capabilities Approach and its Potential for Theorizing Support for Self-Management

### A Capabilities Approach: Introduction to Key Ideas

A capabilities approach is an approach to thinking about how advantaged or disadvantaged people are, or to considering the quality of their lives [42, 57]. In contrast with approaches that focus on how much money people have or how happy they are, capabilities approaches focus on what people can be and do [46]. More particularly, they focus on what it matters that people can be and do (their valued functionings), and whether individual people have the freedoms or genuine opportunities to be and do those things (whether they have the capabilities to realise those valued functionings) [42, 47].

Capabilities approaches were developed originally to guide and assess progress on global human development and social justice [42]. There are several different examples of capabilities approaches, most notably those associated with Amartya Sen [46, 47] and Martha Nussbaum [37]. They vary in a number of respects, including, for example: how valued functionings are identified; whether they focus on certain key capabilities and questions of whether everyone has these to at least a sufficiency level, or on a broader spectrum of capabilities and questions of how well these are achieved across a broader range of levels; and what social theories are drawn on to explain what influences a person’s capabilities [43]. An orientation to questions about what people can be and do, however, is central to all variants [43].

We cannot provide here a full account of capabilities approaches or debates about their theoretical basis, implications and applications. We suggest that a variant of a capabilities approach with the following characteristics will be particularly useful for developing an account of the purpose and means of support for self-management:

- A pluralistic and somewhat open ended view about which capabilities matter. Attention can be paid to both what people generally value being able to be and do (e.g. being well nourished, able to read, respected and able to participate in social activities) and what particular individuals value being able to be and do (e.g. to develop a particular career, support a particular cause, pursue a particular hobby). Some limits may be set, however, on which of the more idiosyncratic personally valued capabilities are supported—e.g. by excluding capabilities to engage in sociopathic behaviour or requiring that more esoteric values can somehow be recognized by others as reasonable (e.g. [46, 47]).
- A strong sense of people as active agents, including of their own capability development [9, 10], but with recognition that people’s agency can be moderated by a variety of factors, including social structures and relationships (see next point).
- A recognition that capabilities are not just bodily or intellectual abilities, but are better understood as genuine opportunities that depend on a combination of more ‘internal’ capacities and more ‘external’ circumstances including access to resources, material environments and social relationships [37, 42, 43, 46, 47]. This ‘relational’ view of capabilities, including capabilities for autonomy [31, 49], encourages attention to the complex ways that particular people and their natural and social environments (including social relationships) interact to influence what those people value and what they can be and do, including how readily they can convert resources and service provision into valued capabilities and functionings [43, 46, 47].
- (following from the previous point) A recognition that capabilities (and perhaps especially shortfalls in capabilities) are often inter-related and in quite complex ways [57]. For example low literacy can limit opportunities for employment which in turn limits scope to afford some health-promoting self-care opportunities (many of which also depend on high levels of literacy); all of these can reduce capabilities to be and feel respected, and (so) to access or benefit from support from healthcare providers [13].
- Scope to attend carefully to human diversity and critically to dynamic changes in the levels and security of people’s capabilities—as well as the way people value particular capabilities—over their life course [43, 57] and so in the context of incurable and degenerative long-term conditions.

In the next sub-section, we consider how the kind of capabilities approach outlined here can be used to develop a broad conception of support for self-management, noting some of what we think are its theoretical advantages.

### Some Key Features of a Capabilities-Based Account of Support for Self-Management

The version of a capabilities approach outlined above generates a broad and responsive view of the overall purpose of support for self-management: to help people to live (and die) well with their long term conditions by promoting the capabilities and functionings that they value. The emphasis on living well provides a justifiable rationale for the greater breadth of purpose while also allowing for some boundary-setting around the ends to be pursued (for example, via the development of requirements that capabilities are both valued by the individuals concerned and socially recognizable as valuable).

If the purpose of support for self-management is couched in terms of helping to ensure people maintain or develop capabilities for valued functionings, health systems and professionals can both take seriously the important concerns that underpin biomedical interests in disease control and keep those concerns in perspective. Capabilities to be free from avoidable pain and other distressing symptoms, for example, can clearly be salient. When biomedical disease control is in principle possible, capabilities to pursue this can be recognized as important because (and/or to the extent that) disease control contributes to other capabilities or functionings that the person either values more intrinsically or views as ultimately significant for living well. For example, a person may view disease control as a means towards realising capabilities to: remain in valued employment; engage in valued projects; be at home rather than in hospital; participate in meaningful social activities; maintain and develop valued relationships with others; be respected within their communities.

An orientation to help ensure people have genuine opportunities to live well with their long-term conditions should encourage health professionals to find out and in some way be responsive to what matters to each particular person they strive to support. This built-in responsiveness permits and invites health professionals to keep questions about the purpose, means and values of support for self-management (and of healthcare more generally) both on the table and “open”. A capabilities approach can help to provide a framework which might scaffold conversations between clinicians and patients, encouraging movement beyond biomedical goals of disease control and risk reduction but without implying either that these are irrelevant or that anything else is a “mere” matter of individual patient preference.

A capabilities lens can also accommodate (and re-frame the significance of) the burdens as well as the benefits of condition-management strategies, including dietary, exercise, rest and medication regimens. It raises questions about which capabilities particular condition-management strategies demand and which they might undermine or threaten. It will focus attention on people’s capabilities to manage or cope with their conditions in ways that mitigate the negative impacts of those conditions on other capabilities they have reason to value in their lives, including capabilities to collaborate effectively in their care.

Notably a capabilities approach encourages attention to what or who people can be, as well as what they can do. Thus the issues of personal (including role) identity and experiences of self, including of being respected, that clearly matter to people with long-term conditions but that prevailing models of support for self-management tend to neglect, are made readily salient.

A capabilities-based account also encourages a broad and nuanced understanding of how clinicians can support people to live well with their long-term conditions. This is because (among other things) it draws attention to the means by which a person’s capabilities can be enhanced or undermined. For example, a capabilities approach has the theoretical scope to explain how important forms of support for valued ways of being are made possible by and embodied within healthcare relationships. A capabilities-based account can thus promote recognition of the value of supportive relationships as well as supportive interventions.

The emphasis that a capabilities-based account places on each person as an agent whose autonomy and scope for action depend in part on their circumstances, and also as an agent of their own capability development, directs attention in a meaningful way to issues of patient empowerment and recognition of patients as co-producers. It encourages nuanced analyses of these, including in the context of complex social power relationships both within and beyond health service encounters.

At the same time a capabilities approach encourages careful thinking about why some people are less able than others to “convert” healthcare and other resources—including the forms of support health professionals offer—into meaningful capabilities. For instance, education about which foods might be beneficial is (on its own) not helpful to someone who cannot afford them, and a referral to free gym sessions will not suffice to secure a capability to exercise regularly for someone who lacks access to affordable transport and childcare availability, or who has little sense of how regular exercise could help them. A capabilities approach can thus discourage equating the offering of a choice with supporting or respecting autonomy [38]. It takes seriously questions about what options are genuinely available to people and what pressures might influence their decisions and behaviours. So rather than assume that people who have been informed about biomedical perspectives on their conditions but have not followed recommended courses of action and/or not achieved biomedical targets have “chosen” not to, or lacked the necessary willpower, a capabilities-based account of support for self-management could help health professionals recognize that what people end up doing or being may be significantly influenced by the circumstantial shaping of their overall life priorities and capabilities.

Overall, a capabilities-based account can help to underpin and inform broader views of the purpose and means of support for self-management by encouraging critical reflection on which of the things that health policies and practices could support patients to be and do “really” matter, and why they matter—not just in general but to each particular person. It generates a more expansive and dynamic evaluative space than many current operational formulations of both (health-related) quality of life and (psychological) wellbeing, and one that can encourage attention to the full range of potential benefits and harms (including for capabilities for autonomy and other aspects of person-al wellbeing [17]) that can be generated during the delivery of care.

## Discussion

We have offered a potentially important explanation for the extent to which the support for self-management that health services and professionals offer people with long term conditions can fall short of the multiple policy ambitions associated with the concept, especially in domains associated with respecting and enabling patients as human moral agents. We have argued that the problem is rooted in part in conceptual-theoretical limitations. When support is oriented to what might be done about risk reduction and disease control, particularly via instructional and motivational means, it tends to reflect and perpetuate a limited view of the person with long-term conditions and of the significance of human relationships.

When narrow views of the purpose or means of support are embedded in healthcare policies, systems and practices, even if they are not explicitly articulated, they tend to: encourage a focus on what biomedicine might influence rather than what illness or dis-ease is experienced; restrict the pursuit of meaningfully respectful and enabling partnership working and broader wellbeing; run the risk of undermining patients’ self-evaluative attitudes, personal identities and autonomous agency (and then of failing to notice those as significant harms); limit recognition of the value of aspects of relationships with clinicians that patients can experience as supportive; and obscure some important practical tensions that clinicians face in the delivery of support for self-management in practice.

We have argued that explicitly broad views of the purpose as well as the means of support for self-management are needed if policy ambitions for the concept are to be fulfilled. We have suggested that enabling people to live (and die) well with their long-term conditions is a promising start for a broad account of purpose, and we have outlined how a capabilities approach could be used to fill out an account from this starting point—or otherwise provide the necessary breadth and overcome the concerns identified with narrow accounts.

We have made a case that a capabilities-based account of support for self-management would be compatible with the pursuit of wellbeing and quality of life in their broadest senses, and via respectful and enabling experiences of health care. It could attend to all aspects of people’s lives and all the various forms of support that people can value in relationships with clinicians, and it could also recognize and help discourage the kinds of undermining of patients’ self-evaluations, identities and scope for autonomous agency that have been reported in some contemporary approaches to support for self-management.^7^

Of course, we are not suggesting that adopting a broader conception of support for self-management, such as that offered by a capabilities-based account, will solve all the challenges of conceptualizing and planning health service support for people with long-term conditions. Several important practical questions remain. First, there are questions about the appropriate scope, balance and prioritization of activities for healthcare professionals in supporting people with long-term conditions. Health professionals have culturally and organizationally circumscribed knowledge bases, social authority, and institutional accountabilities and it is important to consider how they can avoid dissipating their effectiveness by working beyond what they know best (typically biomedicine). This needs careful consideration, but a capabilities-based conception of support for self-management could at least (a) be used to argue that health professionals need to co-ordinate their efforts with those of other people and services to ensure patients can access the support they need to live well with their long-term conditions and (b) help professionals to recognize and avoid the kinds of harm they can cause if they pursue biomedical goals with insufficient attention to people’s other valued (and socially shaped) capabilities.

Second there are questions about the potential tensions in the pursuit of the multiple capabilities that can matter. This includes not only tensions between what health systems or professionals generally recommend and what particular patients want to prioritize, but also tensions between a patient’s various interests and priorities (e.g. for their shorter and longer-term futures and for their concerns for themselves and for other family members). A capabilities approach will not resolve these, but can facilitate a necessary recognition of the complexity of healthcare provision and the problems that need to be overcome in designing and defending a reasonable approach to support for self-management.

Overall we argue that a re-formulation of the concept of support for self-management is needed to improve both the theory and the practice of service provision for people with long-term conditions. A successful reformulation would help expand ideas not just about “support for what” but also “support in what forms”.

The emphasis on support for self-management, and other policies associated with ‘person-centred care’ and concerns to respect and enable people as human moral agents, demand that healthcare professionals work beyond the boundaries of biomedicine. As they are asked to step further beyond these and engage more fundamentally with the complexity of human values and social arrangements, a broadly re-formulated concept of support for self-management, with a sufficiently strong theoretical basis might help to chart the terrain and guide the journey. We have suggested that a capabilities approach provides a rich theoretical resource that may have transformative potential for the formulation of policy and practice relating to support for self-management, and for so-called ‘person-centred’ efforts to ensure health care more generally helps to enable people to live well.

## Footnotes

1 The Institute of Medicine and this paper focus on the support given to people with long-term conditions by health professionals working in formal health services. We acknowledge that family members, friends and particularly ‘peers’ with experience of living with long-term conditions are often hugely important in supporting people to manage (with) their conditions, and in some respects, our points apply to these supporters too. However, the social positions and roles of health professionals generate some particular considerations, so we focus particularly on these.

2 We recognise concerns that support for self-management can be seen as a form of governmentality and/or as primarily oriented to cost-cutting in public services, and also concerns that support for self-management will not in practice achieve the kinds of cost saving that strategists hope for. We set these aside for now as we focus on questions about its potential to deliver the more positive gains in healthcare experience, health and wellbeing that are aspired to in policy rhetoric.

3 Both patient-centred and person-centred care are complex constructs that have been variously defined (see [17]). At core, their concern is to ensure patients are treated appropriately ‘as persons’—so roughly that they are respected and enabled as humans and moral agents [17].

4 Not all of these means of support fall within the usually accepted scope of health system action, although the problems they seek to address are relevant to health system goals.

5 These broader conceptions can also, arguably, respect and enable health professionals as human moral agents, but we do not have space to make that case here.

6 The inclusion of “(and die)” reflects the fact that long-term conditions are incurable. It provides scope to encourage/permit appropriate shifts to palliative goals.

7 We note that Jennifer Prah Ruger and Sridhar Venkatapuram have both made important contributions to thinking about health policy using a capabilities approach. Ruger focuses on two capabilities (to avoid premature death and escapable morbidity) as she makes the case for universal access to healthcare and more distributed forms of healthcare governance [44]. This focus removes the benefits that we derive from a more pluralistic capabilities-based account of living well. Venkatapuram develops an account of health justice based on a consideration of health as a socially influenced meta-capability that is also a means to achieving other capabilities [54]. His account is more compatible with ours, but for our purposes it is important to consider the capabilities associated with living well more generally (and to avoid narrowing these to a health-related subset): we are particularly keen to highlight the ways in which health care can have important implications beyond health.
